# Basophil activation test as predictor of severity and threshold of allergic reactions to egg

**DOI:** 10.1111/all.15875

**Published:** 2023-09-08

**Authors:** Suzana Radulovic, Ru‐Xin Foong, Irene Bartha, Andreina Marques‐Mejias, Marta Krawiec, Matthew Kwok, Zainab Jama, Faye Harrison, Cristian Ricci, Gideon Lack, George Du Toit, Alexandra F. Santos

**Affiliations:** ^1^ Department of Women and Children's Health (Pediatric Allergy), School of Life Course Sciences, Faculty of Life Sciences and Medicine King's College London London UK; ^2^ Children's Allergy Service Evelina London Children's Hospital, Guy's and St Thomas' Hospital London UK; ^3^ Peter Gorer Department of Immunobiology, School of Immunology and Microbial Sciences King's College London London UK; ^4^ Africa Unit for Transdisciplinary Health Research (AUTHeR) North‐West University Potchefstroom South Africa

**Keywords:** baked egg allergy, basophil activation test, egg allergy, severity, threshold

## Abstract

**Background:**

Identifying patients at risk of severe allergic reactions and/or low threshold of reactivity is very important, particularly for staple foods like egg.

**Methods:**

One hundred and fifty children underwent double‐blind placebo‐controlled food challenge (DBPCFC) to baked egg (BE), skin prick testing and blood collection for serology and basophil activation test (BAT). Patients who passed BE DBPCFC underwent loosely cooked egg (LCE) DBPCFC. Severity of allergic reactions was classified following Practall guidelines and threshold dose was determined during DBPCFC.

**Results:**

Sixty out of 150 (40%) children reacted to BE and 16 out of 77 (21%) to LCE on DBPCFC. Considering DBPCFC to BE, 23 children (38%) had severe reactions and 33 (55%) reacted to 0.13 g or less of egg protein (low threshold group). Two children (2 out of 16 = 12%) had severe reactions to LCE. Demographic, clinical and most immunological features were not significantly different between severe/non‐severe BE reactors or low/high threshold groups. Severe BE reactors had higher ovomucoid‐sIgE (*p* = .009) and higher BAT to BE (*p* = .001). Patients with lower threshold to BE had higher IgE‐specific activity (*p* = .027) and BAT to egg (*p* = .007) but lower severity score (*p* = .008). Optimal cut‐offs for ovomucoid‐sIgE had 100% sensitivity, 35% specificity and 60% accuracy and for BAT 76% sensitivity, 74% specificity and 75% accuracy to identify BE severe reactors. Optimal cut‐offs for specific activity had 70% sensitivity, 68% specificity and 69% accuracy and for BAT 70% sensitivity, 72% specificity and 71% accuracy to identify low threshold patients.

**Conclusions:**

BAT was the best biomarker to predict severity and threshold of allergic reactions to BE and can be useful when making decisions about management of egg allergy.

## Graphical abstract

Study participants had DBPCFC to baked egg and, if this was negative, also DBPCFC to loosely cooked egg—patients who developed allergic reactions during DBPCFC were included for analyses. No demographic or clinical features were statistically significantly different between severity or threshold groups. BAT was the best biomarker to identify patients who developed severe reactions or reacted to less than 0.13 g of egg protein.Abbreviations: AUC, area under the curve; BAT, basophil activation test; BAS, basophil; DBPCFC, double‐blind placebo‐controlled food challenge; KU/L, kilounits per litre; OVM, ovomucin; SI, stimulation index; sIgE, specific IgE; sens, sensitivity; spec, specificity; tIgE, total IgE.

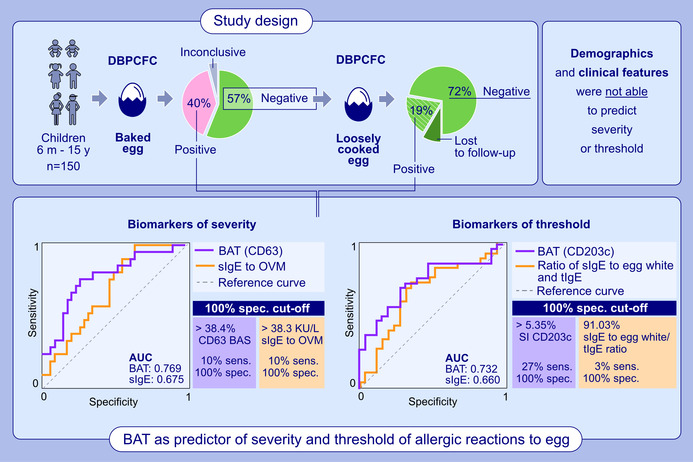

**Abbreviations**
AUC: area under the curve
BAT: basophil activation test
BAS: basophil
DBPCFC: double‐blind placebo‐controlled food challenge
KU/L: kilounits per litre
OVM: ovomucin
SI: stimulation index
sIgE: specific IgE
sens: sensitivity
spec: specificity
tIgE: total IgE

## 1 INTRODUCTION

Food allergy negatively affects the lives of allergic children and their families limiting the diet and causing anxiety and social restrictions due to the need to avoid the allergen and the absence of a curative treatment.^1^ This is particularly impactful for allergy to staple foods such as egg. Given its ubiquity, accidental reactions and possible severe reactions are a constant concern.

Most egg allergic children are tolerant to baked egg and consumption of baked egg has been linked to a better prognosis in terms of spontaneous resolution of egg allergy over time.^2^, ^3^ However, at the time of diagnosis, if allergic patients are not consuming baked egg at all or are only consuming very small amounts, a risk assessment needs to be made to support the decision about introduction of baked egg in the diet and the best setting to perform this to ensure safety.^4^

To address patients' and families' concerns about risk of severe reactions and to better risk stratify egg allergic patients, it would be useful to identify biomarkers that could be used to support decision‐making. In this study, we analyzed demographic, clinical and immunologic characteristics of BAT2 study participants who reacted to baked egg and loosely cooked egg during double‐blind placebo‐controlled food challenges (DBPCFC) to identify biomarkers of severity of allergic reactions and of low threshold dose of reactivity.

## 2 METHODS

### 2.1 Study population

Participants in the BAT2 study (registered at ClinicalTrials.gov with NCT03309488) were prospectively recruited from specialized tertiary Paediatric Allergy clinics in London.^5^ Participants were referred by different clinicians and a screening telephone visit was undertaken to confirm eligibility. Study was approved by the Sponsor, the Research Ethics Committee and the UK Health Research Authority (REC reference 17/LO/0296) before starting. Adults with parental responsibility signed informed consent and children gave assent and signed an assent form if older than 7 years of age, before any study procedure. Children assessed for possible egg allergy were offered to DBPCFC to baked egg and, if they passed this, to DBPCFC to loosely cooked egg. Children younger than 12 months had open challenges. Here we report the severity and threshold of allergic reactions to baked egg or loosely cooked egg in children who had a positive oral food challenge (OFC). The analyses we undertook with regards to the prediction power of biomarkers was restricted to the subset of participants with a positive OFC.

### 2.2 Study procedures

Children aged 6 months to 15 years had clinical assessment as well as skin prick test (SPT), blood collection for serology and basophil activation test (BAT) and OFC to baked egg. Children who passed OFC to baked egg underwent OFC to loosely cooked egg. Their parents filled in food frequency questionnaires (FFQ) and a 7‐day food diary. The study procedures were conducted as previously described in a recent publication.^5^ SPT was performed with a metal lancet, egg extract (ALK Abello), raw egg and baked egg slurry using the challenge food, 50% glycerinated saline solution as negative control and 10 mg/mL histamine as positive control (ALK Abello). The weal diameter was measured after 15 min and calculated as the arithmetic mean of two perpendicular diameters including the largest one. Total IgE and specific IgE and IgG4 levels were measured to egg, egg white, ovomucoid (Gal d 1) and ovalbumin (Gal d 2) using ImmunoCAP (Thermofisher). Specific activity was calculated as previously reported^6^ by dividing the level of allergen‐specific IgE by the level of total IgE in KU/L.

### 2.3 Basophil activation test

The BAT was performed as previously described,^5^ using whole blood and 10fold dilutions in Roswell Park Memorial Institute (RPMI) medium from 10,000 to 0.1 ng/mL of egg extract (ALK Abello) or baked egg white (Sigma‐Aldrich) as stimulants. Baked EW was prepared as a single batch by heating in an oven at 180°C for 20 min and stored in small aliquots for single use at −80°C. RPMI medium alone was used as negative control and anti‐IgE (1 μg/mL, Sigma‐Aldrich) and formyl‐methionyl‐leucylphenylalanine (fMLP, 1 μM, Sigma‐Aldrich) were used as positive controls. Basophil activation was measured as the proportion of CD63‐expressing cells or as the stimulation index of mean fluorescence intensity of CD203c following stimulation. CD63‐APC and CD203c‐PE antibodies were used to measure activation and CD123‐FITC, CD203c‐PE, HLA‐DR‐PerCP and CD63‐APC (Biolegend) were used to identify the basophil cell population by flow cytometry using FACS Fortessa with FACSDiva software (BD Biosciences) to acquire and FlowJo software (version 7.6.1; TreeStar) to analyze the data.

### 2.4 Oral food challenges

All participants underwent DBPCFC to baked egg, except for infants (i.e. children younger than 12 months of age) who had open OFC to baked egg.^5^ The dosing schedule adopted for the baked egg OFC is indicated in Table S1. OFC assessed as high risk were given additional lower starting doses and intravenous cannulation was considered. The criteria to guide the decision to consider an OFC high risk was based on our collective clinical experience of conducting challenges on the NHS and as part of large clinical trials and is shown in Table S2. The outcome of the OFC was determined according to the Practall guidelines (Figure S1). The severity of allergic reactions was classified in real‐time by the clinical team conducting the OFC following the Practall guidelines (Table 1). Peak severity during the OFC overall was recorded, rather than the severity at the time of calling the OFC positive, although most symptoms developed at that time for the majority of patients. The threshold dose for each patient was measured as the cumulative dose tolerated by the time of onset of the allergic reaction. The time between the ingestion of the eliciting dose and the start of symptoms was recorded as was the medication used to treat allergic reactions.

**TABLE 1:** Symptoms and signs developed during oral food challenges to baked egg and to loosely cooked egg performed as part of the BAT2 study and their severity, according to the Practall guidelines. Severity criteria are also summarized and the proportion of patients who fulfilled the severe and non‐severe symptom criteria during oral food challenges to either baked egg or loosely cooked egg are indicated.

Symptoms and signs developed during oral food challenges (OFC)		Criteria for		OFC to baked egg		OFC to loosely cooked egg	
		Severe symptoms/signs	Non‐severe or no symptoms/signs	Number of patients with severe symptoms/signs *n* (%)	Number of patients with non‐severe or no symptoms/signs *n* (%)	Number of patients with severe symptoms/signs *n* (%)	Number of patients non‐severe or no symptoms/signs *n* (%)
Skin	Erythematous rash	>3	≤3	6 (10%)	54 (90%)	0	1 (6%)
	Pruritus	3	0,1,2	0	60 (100%)	0	4 (25%)
	Urticaria/angioedema	3	0,1,2	1 (2%)	59 (98%)	0	7 (44%)
	Rash	3	0,1,2	1 (2%)	59 (98%)	0	4 (25%)
Upper respiratory	Sneezing/itching	3	0,1,2	3 (5%)	57 (95%)	0	8 (50%)
Lower respiratory	Wheezing	1,2,3	0	5 (8%)	55 (92%)	0	0
	Laryngeal	2,3	0,1	3 (5%)	57 (95%)	0	2 (12%)
Gastrointestinal	Subjective complaints	3	0,1,2	4 (7%)	56 (93%)	1 (6%)	4 (25%)
	Objective complaints	2,3	0,1	4 (7%)	56 (97%)	0	1 (6%)
Cardiovasc neurologic		1,2,3	0	2 (3%)	58 (97%)	1 (6%)	0
Overall number of patients in each group				23 (38%)	37 (62%)	2 (12%)	14 (88%)

### 2.5 Statistical analyses and statistical power assessment

Categorical variables were expressed as proportions and compared with Fisher's exact test or chi‐squared test. Quantitative variables were expressed as median and interquartile range and compared using Mann–Whitney *U* test. To assess the discriminative ability of biomarkers to predict severity and threshold categories, receiver operator characteristic (ROC) curve analyses were performed. Optimal cut‐offs were identified based on the Youden index, and sensitivity, specificity and predictive values were calculated from contingency tables for each biomarker based on the outcome of DBPCFC. All statistical evaluations were performed by SPSS version 27. Statistical tests were two tailed and Type‐I error rate was set to 5% (*α* = .05).

The investigation of sample size adequacy was performed by the power.roc.test function from the pRoc package using the R software version 4.0.2. According to our calculation, 50–60 cases and an equal number of controls would determine a Type‐II error rate (probability of false‐negative) below 5% for a ROC curve having an AUC of 0.75 or above. The probability of Type‐II error for a ROC curve having an AUC of 0.70 would be limited to 10%–20%. The above calculations were performed considering a Type‐I error rate (probability of false‐positive) in a range of 1%–5%.

## 3 RESULTS

### 3.1 Characterization of allergic reactions developed during DBPCFC to baked egg and loosely cooked egg as part of the BAT2 study

Sixty (40%) children who underwent OFC to baked egg as part of the BAT2 egg study developed allergic reactions during OFC. Twenty‐three (38%) developed severe allergic reactions, as per the Practall classification. Table 1 describes the signs and symptoms presented during the positive DBPCFC. The median time between ingestion of the culprit dose and the development of symptoms was 15 min (IQR, 5–23). The median eliciting dose was 0.3 g (IQR, 0.1–0.6) and the median cumulative tolerated dose was 0.13 g (IQR, 0.03–0.44) of egg protein. Thirteen (22%) patients were treated with intramuscular adrenaline and three required 2 doses. Table 2 lists the therapies administered to treat allergic reactions during OFC to baked egg and to loosely cooked egg.

**TABLE 2:** Treatments administered during oral food challenges to baked egg performed as part of the BAT2 study.

Medication	Positive OFC to baked egg (*n* = 60)	Positive OFC to loosely cooked egg (*n* = 16)
Adrenaline (intramuscular)	13 (22%)	2 (12%)
1 dose	10 (17%)	2 (12%)
2 doses	3 (5%)	0 (0%)
Anti‐histamines H1 or H2 (systemic)	60 (100%)	15 (94%)
Corticosteroids (systemic)	10 (17%)	0 (0%)
Salbutamol (inhaled)	7 (12%)	0 (0%)
Oxygen	7 (12%)	2 (12%)
Intravenous fluids	1 (2%)	0 (0%)

Sixteen (21%) patients challenged to loosely cooked egg developed allergic reactions. Three (19%) of these had been considered high‐risk challenges, as opposed to 16 (26%) of patients with negative OFC to loosely cooked egg (*p* = .398). Two reactions (12%) to loosely cooked egg were considered severe according to Practall: One patient had significant abdominal pain and the other had neurological symptoms, in combination with symptoms in other organs or systems in both cases (Table 1). These two patients required treatment with intramuscular adrenaline (Table 2). The median time to reaction after the eliciting dose was 13 (IQR, 3–57) minutes. The median threshold of reactivity for patients who reacted to loosely cooked egg was 5.43 g (IQR, 1.18–7.69).

Table S3 reports symptoms developed by and use of adrenaline for treatment of individual patients who reacted to either baked egg or loosely cooked egg.

### 3.2 Demographic and clinical features did not differ between severity or between threshold groups

We compared demographic and clinical features between severe and non‐severe reactors and between allergic patients who reacted to the median cumulative tolerated dose (0.13 g of egg protein) or less and allergic patients who reacted to more than 0.13 g of egg protein during OFC to baked egg. There were no statistically significant differences in any of the demographic and clinical characteristics compared across groups (Tables S4 and S5). Specifically, for instance for severity, there were no differences in age, gender, ethnicity, prior history of consumption of baked egg or prior history of reacting to baked egg, whole egg or raw egg, the type of symptoms developed in previous reactions to egg or co‐existence of atopic comorbidities. The proportion of challenges considered high risk prior to the challenge was the same across severity (*p* = 1.0) and across threshold groups (*p* = 1.0). Interestingly, patients in the lower threshold group had less severe reactions (*p* = .008).

### 3.3 Immunological biomarkers of severity and threshold of allergic reactions during oral food challenges to baked egg

**FIGURE 1:**
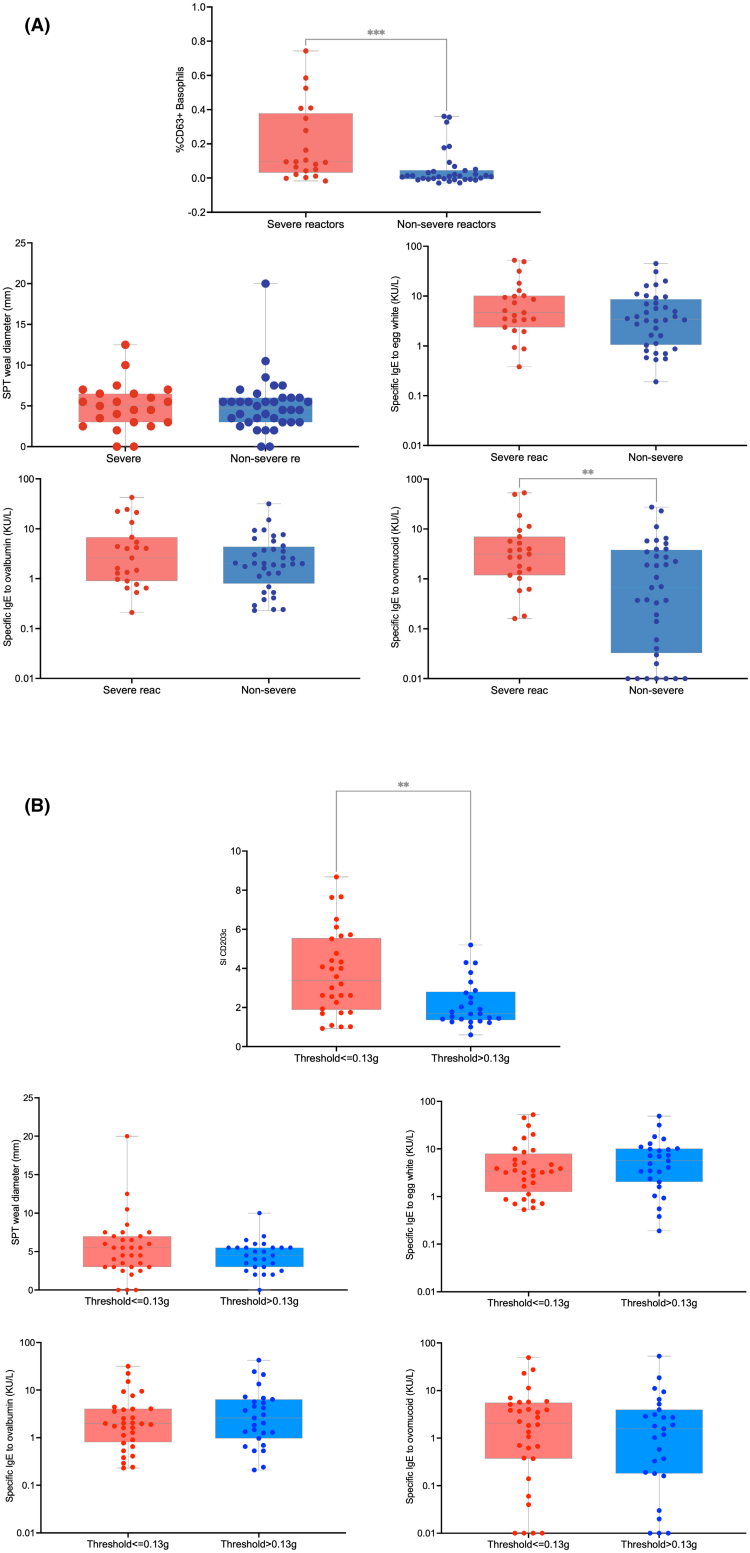
Main immunologic biomarkers in (A) severe versus non‐severe reactors and (B) lower versus higher threshold than 0.13 g of egg protein—results are shown for: basophil activation test (using %CD63+ basophils following stimulation with baked egg white for severity in Figure A and using SI CD203c following stimulation with 10 ng/mL of egg extract for threshold in Figure B), skin prick test to egg white extract, specific IgE to egg white, specific IgE to ovalbumin and specific IgE to ovomucoid.

We then compared severe versus non‐severe reactors and patients with lower versus higher threshold of reactivity to baked egg with regards to immunological markers (Table 3 and Figure 1). There were no statistically significant differences in SPT to egg white extract, raw egg or baked egg; in specific IgE to egg, egg white or OVA; in specific IgG4 or IgG4/IgE ratios to egg, egg white, OVM or OVA, with regards to either severity or threshold.

**TABLE 3:** Immunological features in baked egg allergic children.

A. Severe versus non‐severe reactors during oral food challenge to baked egg			
Immunological characteristics	Severe reactors (*n* = 23)	Non‐severe reactors (*n* = 37)	*p* Value
SPT to egg white extract (mm)	5 (3; 6.5)	4.5 (3; 6)	.772
SPT to raw egg (mm)	8.5 (8; 10.5)	9.5 (6; 11)	.897
SPT to baked egg slurry (mm)	2 (0; 4)	3 (0.5; 3.5)	.661
Total IgE (kU_A_/L)	257 (67; 1933)	290 (92; 1014)	.816
Specific IgE to egg (kU_A_/L)	4.79 (2.45; 12.60)	3.46 (0.88; 7.61)	.128
Specific IgE to egg White (kU_A_/L)	4.66 (2.36; 10.20)	3.44 (1.05; 8.67)	.186
Specific IgE to Gal d 1 (kU_A_/L)	3.13 (1.18; 7.01)	0.69 (0.03; 3.81)	**.009**
Specific IgE to Gal d 2 (kU_A_/L)	2.60 (0.90; 6.78)	2.01 (0.80; 4.37)	.475
Specific IgG4 to egg (mg/L)	0.17 (0.05; 1.37)	0.23 (0.09; 0.89)	.738
Specific IgG4 to egg White (mg/L)	0.21 (0.02; 1.09)	0.16 (0.06; 0.74)	.895
Specific IgG4 to Gal d 1 (mg/L)	0.05 (0.01; 0.29)	0.03 (0.01; 0.20)	.440
Specific IgG4 to Gal d 2 (mg/L)	0.16 (0.01; 0.94)	0.19 (0.06; 0.77)	.803
IgG4/IgE ratio to egg	15 (4; 34)	40 (9; 164)	.062
IgG4/IgE ratio to egg white	11 (3; 43)	28 (6; 99)	.225
IgG4/IgE ratio to Gal d 1	6 (2; 29)	24 (2; 417)	.181
IgG4/IgE ratio to Gal d 2	20 (5; 52)	39 (8; 251)	.225
BAT to baked egg at 0.1 ng/mL (%CD63+ basophils)	9.2 (1.1; 34.9)	1.0 (0; 4.2)	**.001**
BAT to egg white extract at 0.1 ng/mL (SI CD203c)	1.78 (1.27; 3.56)	1.13 (1.0; 1.62)	**.019**
BAT with no stimulation (%CD63+ basophils)	2.2 (1.5; 2.4)	1.9 (1.4; 3.1)	.539
BAT to anti‐IgE (%CD63+ basophils)	36.6 (19.8; 66.3)	431.0 (17.9; 69.3)	.780
BAT to fMLP (%CD63+ basophils)	37.3 (19.6; 49.7)	44.4 (23.7; 53.1)	.401
BAT CD63 egg 100/aIgE (%)	80 (44; 140)	63 (23; 84)	**.015**
Specific activity EW/total IgE	1.3 (0.5; 5.8)	0.9 (0.4; 3.8)	.384

B. Lower versus higher threshold of reactivity to baked egg during oral food challenge			
Immunological characteristics	Threshold dose ≤ 0.13 g (*n* = 33)	Threshold dose > 0.13 g (*n* = 27)	*p* Value
SPT to egg white extract (mm)	5.5 (3; 7)	4.5 (3; 5.5)	.286
SPT to raw egg (mm)	9.5 (7; 12)	8.5 (7.5; 10.5)	.434
SPT to baked egg slurry (mm)	3.5 (0; 4)	3 (1; 3)	.940
Total IgE (kU_A_/L)	231 (78; 411)	750 (87; 2346)	**.036**
Specific IgE to egg (kU_A_/L)	3.36 (0.88; 5.49)	6.17 (1.87; 11.8)	.171
Specific IgE to egg White (kU_A_/L)	3.42 (1.25; 7.94)	5.64 (2.03; 10.2)	.290
Specific IgE to Gal d 1 (kU_A_/L)	2.08 (0.37; 5.54)	1.57 (0.18; 3.97)	.498
Specific IgE to Gal d 2 (kU_A_/L)	1.99 (0.80; 4.05)	2.60 (0.97; 6.36)	.503
Specific IgG4 to egg (mg/L)	0.23 (0.08; 0.68)	0.18 (0.09; 1.12)	.778
Specific IgG4 to egg White (mg/L)	0.16 (0.06; 0.61)	0.19 (0.04; 1.0)	.945
Specific IgG4 to Gal d 1 (mg/L)	0.04 (0.01; 0.18)	0.05 (0.01; 0.33)	.699
Specific IgG4 to Gal d 2 (mg/L)	0.18 (0.04; 0.67)	0.17 (0.07; 0.83)	.675
IgG4/IgE ratio to egg	31.6 (8.1; 156.0)	19.9 (5.5; 63.5)	.605
IgG4/IgE ratio to egg white	19.1 (6.3; 99.8)	14.3 (3.6; 65.5)	.681
IgG4/IgE ratio to Gal d 1	7.6 (1.7; 106.3)	23.1 (2.9; 416.7)	.215
IgG4/IgE ratio to Gal d 2	37.5 (5.7; 139.8)	22.0 (6.2; 133.4)	.749
BAT to egg white extract at 1 ng/mL (SI CD203c)	1.73 (1.18; 4.11)	1.24 (1.07; 1.65)	**.012**
BAT to egg white extract at 10 ng/mL (SI CD203c)	3.11 (1.73; 5.32)	1.67 (1.27; 2.75)	**.007**
BAT with no stimulation (%CD63+ basophils)	1.8 (1.3; 2.6)	2.2 (1.5; 2.7)	.196
BAT to anti‐IgE (%CD63+ basophils)	39.0 (17.9; 72.7)	32.5 (19.8; 51.9)	.330
BAT to fMLP (%CD63+ basophils)	42.3 (25.4; 55.7)	37.3 (22.2; 50.5)	.403
BAT CD63 egg 0.1/aIgE (%)	6.5 (0.7; 33.8)	2.4 (0; 7.5)	.411
Specific activity EW/total IgE (%)	1.27 (0.71; 5.85)	0.59 (0.36; 2.29)	**.027**

*Note*: *p* Values refer to Mann–Whitney *U* test.

Statistically significant *p* values are indicated in bold.

Abbreviations: anti‐IgE, anti‐immunoglobulin E; BAT, basophil activation test; EW, egg white; Ig, immunoglobulin; SPT, skin prick test

The only biomarkers that were statistically significantly different in severe reactors compared to non‐severe reactors were specific IgE to OVM and basophil activation test to baked egg measured with CD63 following stimulation with baked egg white at all concentrations tested from 0.1 to 10,000 ng/mL (and 0.1–1000 ng/mL for CD203c) but not following stimulation with egg extract (Tables 3A and S6 and Figures 1A and S2A).

The only biomarkers that were statistically significantly different in patients with lower threshold of reactivity (at 0.13 g or lower) compared with patients with higher threshold of reactivity (more than 0.13 g) were specific activity of sIgE to egg white and basophil activation measured with CD203c (but not CD63) at the lowest concentrations of baked egg (0.1 ng/mL) or of egg extract (1 and 10 ng/mL)—Tables 3B and S7 and Figure 1B and S2B.

### 3.4 Biomarkers to predict both severity and threshold of reactivity to baked egg during DBPCFC

**FIGURE 2:**
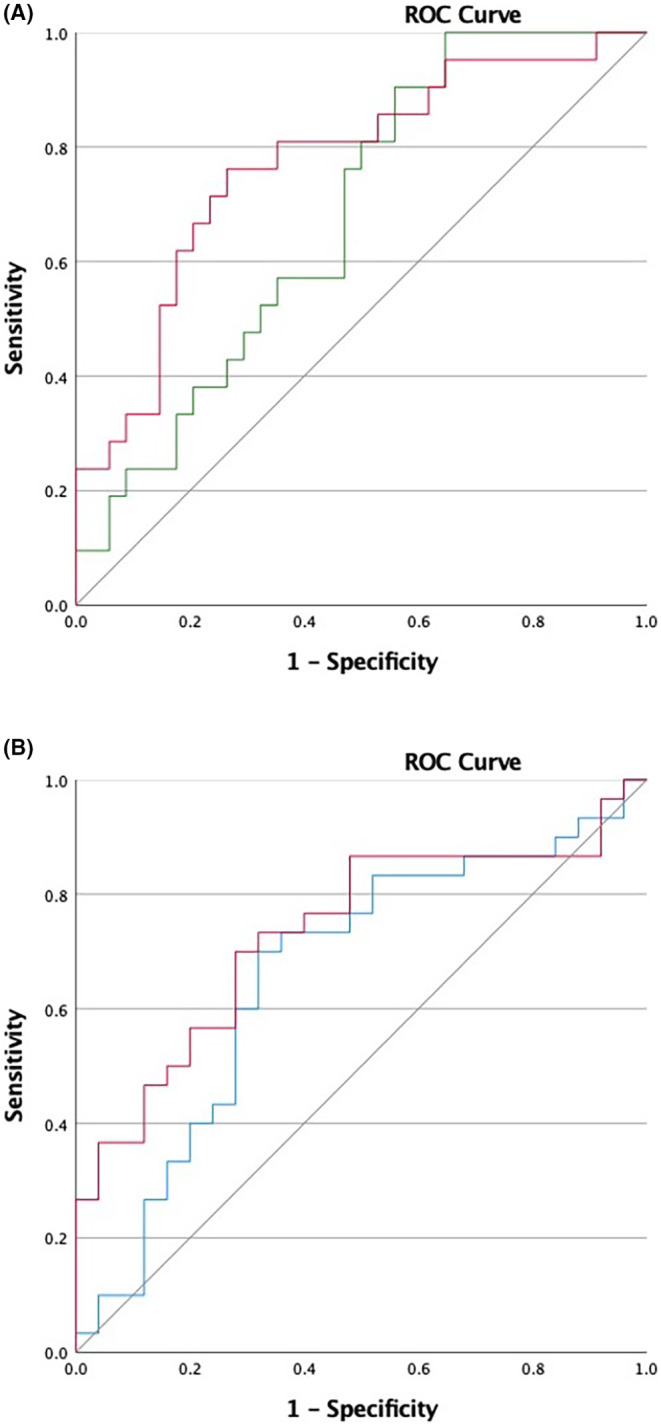
Receiver operator characteristic curve for the best biomarkers of (A) severity and (B) threshold of allergic reactions to baked egg during challenges.

We performed ROC curve analyses for the biomarkers that best discriminated between the severe/non‐severe and the lower/higher threshold groups (Figure 2). The area under the ROC curve were 0.769 (95% CI, 0.638–0.900) for BAT and 0.676 (95% CI, 0.536–0.816) for sIgE to OVM for severity; and 0.732 (95% CI, 0.598–0.866) for BAT and 0.660 (95% CI, 0.511–0.809) for specific activity of sIgE to egg white for threshold. Based on the ROC curves, we calculated optimal cut‐offs based on the Youden index and the cut‐offs with 100% sensitivity and 100% specificity, which are represented in Table 4.

**TABLE 4:** Diagnostic performance of optimal, 100% sensitivity and 100% specificity cut‐offs for the best biomarkers for severity and threshold of allergic reactions to baked egg during oral food challenge.

Diagnostic tests	Cut‐off		AUC ROC	Sensitivity	Specificity	PPV	NPV	Diagnostic accuracy	TP/FP	TN/FN
Severity										
Specific IgE OVM (KU/L)	100% S	0.15	0.677	100%	35%	49%	100%	60%	21/22	12/0
	Optimal									
	100% Sp	38.3	0.548	10%	100%	100%	64%	65%	38/0	0/19
BAT to baked egg white (%CD63, 0.1 ng/mL)	100% S	0	0.544	100%	9%	40%	100%	44%	21/31	3/0
	Optimal Youden	4.0	0.749	76%	74%	64%	83%	75%	16/9	25/5
	100% Sp	38.4	0.619	24%	100%	100%	68%	71%	5/0	34/16
Threshold										
Specific activity egg white/total IgE (%)	100% S	0.12	0.520	100%	4%	56%	100%	56%	30/24	1/0
	Optimal	0.94	0.690	70%	68%	72%	65%	69%	21/8	17/9
	100% Sp	91.03	0.517	3%	100%	100%	46%	47%	1/0	25/29
BAT to egg (SI CD203c, 10 ng/mL)	100% S	0.77	0.564	100%	4%	56%	100%	56%	30/24	1/0
	Optimal Youden	2.54	0.710	70%	72%	75%	67%	71%	21/7	18/9
	100% Sp	5.35	0.634	27%	100%	100%	53%	60%	8/0	25/22

Abbreviations: AUC, area under the curve; BAT, basophil activation test; FN, false‐negative; FP, false‐positive; IgE, immunoglobulin E; NPV, negative predictive value; OVM, ovomucoid; PPV, positive predictive value; ROC, receiver operating characteristics curve; S, sensitivity; Sp, specificity; TN, true‐negative; TP, true‐positive.

We have previously reported on the diagnostic performance of BAT to support the diagnosis of baked egg and loosely cooked egg allergy. In Figure S3 we presented a proposed approach to incorporating diagnosis and severity prediction based on BAT results to inform clinical decision‐making, which should consider the clinical and wider context of individual patients.

## 4 DISCUSSION

Identifying the patients at risk of severe symptoms or of reacting to small amounts of the allergen is an important part of the management of food allergic patients. This gains particular importance when managing patients with allergy to staple foods that are commonly encountered and with allergy to foods where consumption of small amounts namely in the baked form is often tolerated and recommended by healthcare professionals, like egg.^4^ We compared the severity of allergic reactions and the cumulative dose tolerated during DBPCFC with various demographic, clinical and immunological markers. Severe and non‐severe groups and lower/higher threshold groups were not different in terms of demographic and clinical parameters. Only immunological markers were able to differentiate the groups: BAT and sIgE to ovomucoid were the best predictor markers of the reaction severity and BAT and egg white–IgE‐specific activity were the best predictor markers of the reaction threshold. BAT was the best biomarker for both severity and threshold.

Our study clearly shows that the BAT is the most effective tool to identify patients at risk of developing severe symptoms to small amounts of baked egg. Similar to our previous observations in peanut allergy,^7^ basophil reactivity, defined as the proportion of activated basophils at various allergen concentrations, was significantly higher in severe than in non‐severe reactors; whereas basophil sensitivity, defined by the allergen concentration at which basophils respond, was more relevant for threshold. The differences in the BAT results between threshold groups were significant only at the lower allergen concentrations, which is interesting. For severity, the differences in BAT were only observed when using baked egg protein and not when using egg extract, which is intriguing and suggests that the differential ability to respond to the heat‐modified proteins is somewhat the key to severity risk. Another difference in using BAT as a marker of severity versus threshold is that, for severity, CD63 was the most informative activation marker and, for threshold, this was CD203c. This contrast intuitively makes sense as CD63 is a marker of anaphylactic degranulation and CD203c is a marker of piecemeal degranulation.^8^ If one tries to visualize the biology of basophil degranulation, following exposure to low concentrations of the allergen, low‐grade activation happens (and patients with low threshold of reactivity show higher basophil activation at this stage) and, as activation progresses, anaphylactic degranulation develops leading to a greater release of pro‐inflammatory mediators, causing more severe symptoms.

It was surprising that patients in the lower threshold group were also in the lower severity group. A possible explanation is that patients who develop symptoms earlier after consuming a lower dose end up consuming less of the allergen and trigger fewer effector cells leading to lower release of mediators and less severe symptoms. Conversely, patients who react to a higher threshold had consumed more of the allergen by the time they reacted leading to a greater amount of allergen present in their body and to a greater release of mediators by mast cells and basophils and more severe symptoms.

We had previously reported data on BAT as predictor of severity and threshold of allergic reactions to peanut in independent cohorts of children being assessed for possible peanut allergy.^7^, ^9^ In the BAT2 study, we have confirmed this for egg allergy. Our findings are particularly useful in children, a population in which egg allergy is most common and for whom egg represents a valid and economic source of high‐quality nutrients.^10^, ^11^ The relevance of our findings needs to be further investigated in adults.

Our methodology was very robust; for instance, the BAT2 study was a diagnostic study designed according to the STARD guidelines,^12^ in which patients were prospectively and sequentially recruited by various clinicians in specialized Paediatric Allergy clinics. It is therefore a representative population of the patients seen at the secondary and tertiary care levels. However, results may not be applicable to the general population. All participants were submitted to the reference standard, oral food challenge and this was DBPCFC in all children aged 12 months or above.

The severity of allergic reactions was classified in real time by the clinical team assessing the patients during the DBPCFC using pre‐defined criteria. Threshold was carefully measured based on the doses that were eaten and tolerated by the patients during the challenges by specialized dietitians. Similar to a previous study on peanut allergy,^7^ we selected the population of children who had a positive OFC to baked egg or loosely cooked egg as part of the BAT2 study to look for predictive factors of high risk.^5^ Patients with a negative OFC, who were excluded from these analyses, could have been allergic patients with a high threshold and could have reacted to a higher dose of egg than the cumulative dose used in our challenge protocol. However, the dose that we used (Table S1) was quite high and higher than the doses used in many studies and clinical settings, as we wanted to be sure it was safe to advise consumption of baked egg in children who tolerated baked egg and reacted to loosely cooked egg. Furthermore, we used doses tailored for the different age groups and therefore the doses are likely to be relevant to the portion sizes that children would consume in day‐to‐day life.

The fact that the proportion of challenges considered high risk by the clinical team before the challenge was very similar in patients who had more severe reactions and in patients who had less severe reactions attests to our current inability to stratify patients' risk with precision,^13^, ^14^ even in optimal conditions of an in‐hospital supervised and controlled allergen exposure like in an OFC. In a real‐life setting, there other variables that can affect the severity of allergic reactions^15^ and can further confound the prediction of risk.^16^ Interestingly, there was no difference in the type of symptoms developed in the last reaction to baked egg in patients who reacted severely to baked egg during DBPCFC and the ones that reacted with non‐severe manifestations. This is consistent with previous reports from fatal and near‐fatal case series^17^, ^18^ and with a recent rapid systematic review of the literature with meta‐analyses,^13^ that suggest that previous severity of allergic reactions does not necessarily reflect severity of future reactions.

The discrepancy between the severity of allergic reactions historically versus witnessed as part of DBPCFC is probably due to the multitude of factors that influence reaction severity,^15^ many of which depend on the circumstances in which the reaction occurs, and also possibly recall bias when severity is measured based on reported symptoms rather than objective signs recorded by a medical team and supported by medical examination like in a DBPCFC. It was interesting to see the lower proportion of patients with severe reactions to loosely cooked egg compared to the proportion of patients with severe reactions to baked egg, which probably reflects the more benign phenotype of patients tolerating baked egg who were later challenged to loosely cooked egg (as opposed to patients who reacted to baked egg and were not challenged to loosely cooked egg) and also possibly the time gap between baked egg and loosely cooked egg challenges, during which patients were consuming baked egg in the diet. This could also explain the much higher cumulative threshold observed in patients who reacted to loosely cooked egg compared with patients who reacted to baked egg during challenges.

The median threshold of reactivity between baked egg reactors and loosely cooked egg reactors is another element of the better prognosis of egg allergic patients who tolerate baked egg and possibly result from the immunomodulatory effect of baked egg consumption.^2^, ^19^ It was surprising to see some patients with negative SPT to egg white extract and baked egg (*n* = 2 and *n* = 6, respectively, but not raw egg) having severe reactions during the baked egg OFC. Our findings suggest that BAT or at least specific IgE to egg white should be done before advising about reintroduction of egg in the diet of egg allergic children and that such advice should not be based on SPT alone. Previous studies have associated ovomucoid‐specific IgE with baked egg allergy and persistent egg allergy^20^, ^21^, ^22^, ^23^, ^24^, ^25^, ^26^, ^27^, ^28^, ^29^; however, there is no evidence that it could be responsible for more severe allergic reactions. In the BAT2 study ovalbumin‐specific IgE was more informative diagnostically than ovomucoid‐specific IgE, as previously reported.^5^

A limitation of our study was the fact that patients with a definite clinical diagnosis of egg allergy, based on the history and highly predictive IgE sensitization test results, were not referred to the study, possibly restricting the degree of severity of allergic reactions observed; however, it would be ethically questionable to challenge them. It would also be ethically questionable to challenge loosely cooked egg patients who had reacted to baked egg as baked egg is less allergic due to lower dose, extensive heating and matrix effect.^30^ While it is known that about 80% of egg allergic patients tolerate baked egg,^2^ the case of a patient who reacts to baked egg and not loosely cooked egg is yet to be reported. For this reason, we decided to have the baked egg challenge first and only challenge to loosely cooked egg patients who tolerated baked egg; however, this design had the potential to affect the severity assessments and performance of biomarkers. Of note, in the BAT2 study, we did challenge patients with high levels of sensitization who did not have a history of reaction but who would not have been challenged in daily clinical practice due to perceived risk and/or limited capacity for OFC. Additional selection bias was minimized by a sequential recruitment scheme which should result in a sufficient degree of randomness of the sample. A good level of representativeness of the target population can be assumed as our study population is derived by numerous independent clinicians. In terms of sample size, we cannot exclude the presence of false‐negative results for the sample size adopted here may have limited our study. The study was powered to look at diagnosis and only a fraction of the population reacted during the challenges, although the proportion of positive baked egg challenges in the BAT2 study was higher (40%) than often observed in the routine clinical setting (20%).^31^ However, we showed that, with the current sample size, any possible issue due to lack of statistical power would arise only for AUC below the value of 0.7, which is not a value of clinical interest. Finally, we cannot exclude a possible bias given by the residual confounding from unknown factors. However, the small heterogeneity of our sample points out for a limited residual confounding effect. Further validation is required with more studies adopting such a rigorous approach and being based on different patient populations should be conducted to investigate the reliability and generalizability of our results.

We have recently shown that BAT is a very good diagnostic marker for egg allergy, particularly in the younger age group (younger than 2 years) and in patients already consuming some baked egg in the diet.^5^ We could use BAT further to inform practical advice given to egg allergic patients about consumption of baked egg and to identify patients at high‐risk of a severe reaction, who would benefit from an in‐hospital challenge or avoidance, depending on patient's preferences. Given the multitude of factors that can influence the severity of allergic reaction, decision should not be made on BAT alone but rather in combination with other elements, in the wider context of the patient in question. With the progression from avoidance to active management of food allergies, having more precise biomarkers to support clinical decision‐making would be very helpful, particularly for foods that are ubiquitously present in the diet and for allergies that are often transient like egg allergy. The introduction of baked egg in the diet can make a significant difference in the quality of the diet and in the quality of life of children and their families and may define a better prognosis for future resolution of egg allergy, although this is still a matter of debate. In any case, BAT can support the current changes in clinical practice and, most importantly, can support clinical decision‐making, especially when immunomodulatory treatments or re‐introduction of baked egg in the diet are being considered.

## AUTHOR CONTRIBUTIONS

AM‐M, SR, R‐XF, IB, MK, GDT and AFS performed study procedures related to patient recruitment and cared for study participants. MKw and ZJ performed and analyzed the basophil activation test. FH and AFS designed the food frequency questionnaires and oral food challenge protocols. CR performed the statistical analyses. GDT and GL critically reviewed the study protocol. AFS designed the study protocol and acted as chief investigator for the study, obtained and managed the research funding, supervised data acquisition, data management and data analyses, and wrote the first version of the manuscript. All authors critically reviewed the manuscript and approved its final version.

## FUNDING INFORMATION

This study was funded by the Medical Research Council through MRC Clinician Scientist Fellowship MR/M008517/1 and MRC Transition Fellowship MR/T032081/1 awarded to A. F. Santos.

## CONFLICT OF INTEREST STATEMENT

Dr Radulovic reports salary support from grants from National Institute of Allergy and Infectious Diseases (NIAID, NIH). Dr Lack reports grants from National Institute of Allergy and Infectious Diseases (NIAID, NIH), other from Food Allergy & Research Education (FARE), other from MRC & Asthma UK Centre, other from UK Dept of Health through NIHR, other from National Peanut Board (NPB), other from The Davis Foundation, during the conduct of the study; shareholder in DBV Technologies, and Mighty Mission Me, personal fees from Novartis, personal fees from Sanofi‐Genyzme, personal fees from Regeneron, personal fees from ALK‐Abello, personal fees from Lurie Children's Hospital, outside the submitted work. Dr Du Toit reports grants from National Institute of Allergy and Infectious Diseases (NIAID, NIH), Food Allergy & Research Education (FARE), MRC & Asthma UK Centre, UK Dept of Health through NIHR, Action Medical Research and National Peanut Board. Scientific Advisory Board member Aimmune. Investigator on pharma‐sponsored allergy studies (Aimmune, and DBV Technologies). Scientific advisor to Aimmune, DBV and Novartis. Dr Santos reports grants from Medical Research Council (MR/M008517/1; MC/PC/18052; MR/T032081/1), Food Allergy Research and Education (FARE), the Immune Tolerance Network/National Institute of Allergy and Infectious Diseases (NIAID, NIH), Asthma UK (AUK‐BC‐2015‐01), BBSRC, Rosetrees Trust and the NIHR through the Biomedical Research Centre (BRC) award to Guy's and St Thomas' NHS Foundation Trust, during the conduct of the study; personal fees from Thermo Scientific, Nutricia, Infomed, Novartis, Allergy Therapeutics, Buhlmann, as well as research support from Buhlmann and Thermo Fisher Scientific through a collaboration agreement with King's College London. The other authors have nothing to disclose.

## Supporting information


Appendix S1

## Data Availability

The data that support the findings of this study are available on request from the corresponding author. The data are not publicly available due to privacy or ethical restrictions.
