# Supplementary material for: Basophil activation test as predictor of severity and threshold of allergic reactions to egg
Source: Allergy. 2023 Sep 8;79(2):419–31. doi: 10.1111/all.15875 (PMC10952485; doi:10.1111/all.15875)

**Basophil activation test as predictor of severity and threshold of allergic reactions to egg**

**Short title:** BAT for severity and threshold of egg allergic reactions

Suzana Radulovic^1,3^*, Ru-Xin Foong^1,2,3^*, Irene Bartha^1,3^*, Andreina Marques-Mejias^1,3^*, Marta Krawiec^1,3^*, Matthew Kwok^1,2^, Zainab Jama^1,2^, Faye Harrison^1,2^, Cristian Ricci^4^, Gideon Lack^1,2,3^, George Du Toit^1,3^, Alexandra F. Santos^1,2,3^

**shared first authors*

^1^Department of Women and Children’s Health (Pediatric Allergy), School of Life Course Sciences, Faculty of Life Sciences and Medicine, King’s College London, London, United Kingdom

^2^Peter Gorer Department of Immunobiology, School of Immunology and Microbial Sciences, King’s College London, London, United Kingdom

^3^Children’s Allergy Service, Evelina London Children’s Hospital, Guy’s and St Thomas’ Hospital, London, United Kingdom

^4^Africa Unit for Transdisciplinary Health Research (AUTHeR), North-West University, Potchefstroom, South Africa

**Supplementary material:**

**Table S1.** Dosing schedule adopted for the challenges to baked egg and lightly cooked egg in the BAT2 study. .*The initial doses will be given only in patients considered to be high-risk (HR). **Cumulative dose does not include the high-risk doses. Abbreviations: DBPCFC: Double-blind placebo-controlled food challenges.

| **Age** | | **6-12 months** | **1-3 years** | **4-10 years** | **11-16 years** |
| --- | --- | --- | --- | --- | --- |
| Type of challenge | | Open | DBPCFC | DBPCFC | DBPCFC |
| Number of placebo doses randomly interspersed | | 0 | 1 | 1 | 1 |
| FOOD | | BAKED EGG | | | |
| Active doses  (g) | High Risk Dose 1* | 0.003 | 0.003 | 0.003 | 0.003 |
|  | High Risk Dose 2* | 0.01 | 0.01 | 0.01 | 0.01 |
|  | Dose 1 | 0.03 | 0.03 | 0.03 | 0.03 |
|  | Dose 2 | 0.1 | 0.1 | 0.1 | 0.1 |
|  | Dose 3 | 0.2 | 0.3 | 0.3 | 0.3 |
|  | Dose 4 | 0.4 | 0.6 | 0.6 | 0.6 |
|  | Open dose | 0.8 | 1.0 | 1.5 | 2.5 |
|  | **Cumulative protein dose**** | **1.53** | **2.03** | **2.53** | **3.53** |
|  | Total number of muffin/cookies | 1.0 | 1.3 | 1.6 | 2.2 |
|  | | | | | |
| FOOD | | LIGHTLY COOKED EGG | | | |
| Active doses  (g) | High Risk Dose 1* | 0.003 | 0.003 | 0.003 | 0.003 |
|  | High Risk Dose 2* | 0.01 | 0.01 | 0.01 | 0.01 |
|  | Dose 1 | 0.03 | 0.03 | 0.03 | 0.03 |
|  | Dose 2 | 0.1 | 0.1 | 0.1 | 0.1 |
|  | Dose 3 | 0.3 | 0.3 | 0.3 | 0.3 |
|  | Dose 4 | 1.0 | 1.0 | 1.0 | 1.0 |
|  | Open dose 1 | 2.0 | 2.0 | 2.0 | 2.0 |
|  | Open dose 2 | 3.0 | 4.0 | 5.0 | 6.0 |
|  | **Cumulative protein dose**** | **6.43** | **7.43** | **8.43** | **9.43** |
|  | Total number of 50g medium eggs | 0.9 | 1.0 | 1.2 | 1.3 |

**Table S2.** Criteria to consider the oral food challenge high-risk according to the study protocol. These criteria served as guidance only and the decision to consider high-risk is not limited to these criteria and left at the discretion of the investigators.

| Criteria to consider high-risk challenge |
| --- |
| - History of severe allergic or FPIES reaction to the food being challenged in the last 12 months - History of anaphylaxis to the challenge food* - Clinical diagnosis of asthma or recurrent wheezing - Hospitalisation or oral steroid rescue therapy required for asthma exacerbation in last 12 months - SPT to egg white ≥8 mm |

*irrespective of whether the challenge is to the baked or the fresh/loosely cooked form

Abbreviations: FPIES: food-protein induced enterocolitis syndrome; Skin Prick Test (SPT).

**Table S3.** Symptoms in individual patients who reacted to either baked egg or loosely cooked egg in the BAT2 study.

| ID | Food challenge | Symptoms developed during challenge | Adrenaline given? |
| --- | --- | --- | --- |
| BAT002 | Baked Egg | oral pruritis, gastro symptoms | no |
| BAT004 | Baked Egg | localized cutaneous erythema, localized urticaria, mild asthma | yes |
| BAT006 | Loosely cooked egg | localized angiodema, gastro symptoms | yes |
| BAT008 | Baked egg | localized urticaria, laryngeal oedema | no |
| BAT013 | Baked egg | localized angiodema, generalized erythema | yes |
| BAT016 | Baked egg | Localized cutaneous erythema, Localized urticaria, Gastrointestinal symptoms, Rhinitis, Laryngeal edema | no |
| BAT018 | Baked egg | oral pruritis, gastrointestinal symptoms | no |
| BAT020 | Baked egg | localized cutaneous erythema, localized angiodema | no |
| BAT023 | Loosely cooked egg | localized cutaneous erythema, oral pruritus | no |
| BAT025 | Baked egg | gastrointestinal symptoms, rhinitis, laryngeal oedema, mild asthma | yes |
| BAT029 | Baked egg | oral pruritis | no |
| BAT030 | Baked egg | localized cutaneous erythema, localized urticaria, gastro symptoms | no |
| BAT033 | Baked egg | gastro symptoms | no |
| BAT035 | Baked egg | Localized cutaneous erythema, Gastrointestinal symptoms | no |
| BAT036 | Loosely Cooked Egg | Localized cutaneous erythema, Localized urticaria | no |
| BAT037 | Baked egg | Gastrointestinal symptoms, Rhinitis | no |
| BAT038 | Loosely cooked egg | localized cutaneous erythema, localized urticaria | no |
| BAT039 | Loosely Cooked Egg | Gastrointestinal symptoms | no |
| BAT040 | Baked egg | generalized urticaria | yes |
| BAT041 | Baked egg | Localized cutaneous erythema, Oral pruritus, Gastrointestinal symptoms | no |
| BAT044 | Loosely Cooked Egg | Localized cutaneous erythema, Localized urticaria | no |
| BAT045 | Baked egg | localized cutaneous erythema, oral pruritis, gastrointestinal symptoms | yes |
| BAT051 | Baked egg | Localized cutaneous erythema, Localized urticaria, Localized angioedema, Gastrointestinal symptoms, Rhinitis | no |
| BAT054 | Baked egg | Localized cutaneous erythema, Oral pruritus | no |
| BAT056 | Baked egg | Oral pruritus, Gastrointestinal symptoms | no |
| BAT057 | Loosely Cooked Egg | Gastrointestinal symptoms | no |
| BAT058 | Baked egg | Gastrointestinal symptoms | yes |
| BAT064 | Baked egg | Localized urticaria, | no |
| BAT065 | Baked egg | Gastrointestinal symptoms, Rhinitis, | no |
| BAT070 | Loosely Cooked Egg | Oral pruritus, Rhinitis | no |
| BAT073 | Baked egg | Localized cutaneous erythema, Localized urticaria, | no |
| BAT074 | Baked egg | Localized urticaria | no |
| BAT077 | Loosely Cooked Egg | Localized cutaneous erythema, Localized angioedema | no |
| BAT078 | Baked egg | Localized cutaneous erythema, Rhinitis, Mild asthma | yes |
| BAT088 | Baked egg | Gastrointestinal symptoms, Rhinitis | no |
| BAT089 | Baked egg | Gastrointestinal symptoms, Rhinitis | no |
| BAT091 | Baked egg | Localized urticaria, Localized angioedema, Gastrointestinal symptoms | no |
| BAT098 | Baked egg | Localized angioedema, | no |
| BAT101 | Baked egg | Oral pruritus, Gastrointestinal symptoms | no |
| BAT110 | Baked egg | Oral pruritus, Gastrointestinal symptoms | no |
| BAT111 | Baked egg | Oral pruritus, Gastrointestinal symptoms, Rhinitis | no |
| BAT119 | Loosely Cooked Egg | Gastrointestinal symptom | yes |
| BAT121 | Baked egg | Localized urticaria, Gastrointestinal symptoms, Rhinitis | yes |
| BAT122 | Baked egg | Oral pruritus, Gastrointestinal symptoms | no |
| BAT123 | Loosely cooked Egg | Gastrointestinal symptoms | no |
| BAT143 | Loosely cooked egg | Oral pruritus, Rhinitis | no |
| BAT144 | Loosely cooked Egg | Localized urticaria | no |
| BAT169 | Baked egg | Localized urticaria, Localized angioedema, Oral pruritus, Generalized erythema | no |
| BAT131 | Baked egg | Oral pruritus, Gastrointestinal symptoms | no |
| BAT179 | Baked egg | Oral pruritus, Gastrointestinal symptoms | no |
| BAT187 | Baked egg | Oral pruritus, Gastrointestinal symptoms, Rhinitis | no |
| BAT191 | Baked egg | Localized cutaneous erythema, Localized urticaria, Localized angioedema | no |
| BAT211 | Baked egg | Oral pruritus | no |
| BAT210 | Baked egg | Localized angioedema, Oral pruritus | no |
| BAT190 | Baked egg | Gastrointestinal symptoms | no |
| BAT192 | Baked egg | Oral pruritus, Gastrointestinal symptoms | no |
| BAT204 | Baked egg | Localized urticaria, Rhinitis | yes |
| BAT199 | Baked egg | Generalized erythema, Gastrointestinal symptoms, Rhinitis | no |
| BAT198 | Baked egg | Localized cutaneous erythema, Localized urticaria, Gastrointestinal symptoms | yes |
| BAT207 | Baked egg | Localized urticaria, Localized angioedema, Rhinitis | yes |
| BAT104 | Loosely cooked egg | Oral pruritus | no |
| BAT224 | Baked egg | Gastrointestinal symptoms, Rhinitis | yes |
| BAT234 | Baked egg | Oral pruritus | no |
| BAT233 | Baked egg | Localized cutaneous erythema, Localized angioedema, Oral pruritus. | no |
| BAT222 | Baked egg | Localized urticaria, Oral pruritus | no |
| BAT219 | Baked egg | Oral pruritus, Rhinitis | no |
| BAT232 | Baked egg | Oral pruritus | no |
| BAT226 | Baked egg | Oral pruritus, Gastrointestinal symptoms, Rhinitis | no |
| BAT220 | Baked egg | Localized urticaria, Oral pruritus, Gastrointestinal symptoms | no |
| BAT212 | Loosely cooked egg | Generalized urticaria, Rhinitis | no |
| BAT216 | Loosely cooked egg | Localized urticaria, Oral pruritus | no |

**Table S4.** Demographic and clinical characteristics of baked egg allergic patients (n=60) who developed severe or non-severe reactions during DBPCFC to baked egg, according to the Practall guidelines. P values refer to Mann-Whitney U test.

| **Demographic and clinical characteristics** | **Severe reactors**  **(n=23)** | **Non-severe reactors (n=37)** | **P value** |
| --- | --- | --- | --- |
| Age (years) | 6.5 (4; 9) | 5.5 (2.5; 8) | 0.616 |
| Gender (% females) | 10 (44%) | 16 (43%) | 1.0 |
| Ethnicity |  |  |  |
| - White | 14 (61%) | 25 (68%) |  |
| - Black | 2 (9%) | 4 (11%) |  |
| - Asian | 3 (13%) | 1 (3%) | 0.377 |
| - Chinese | 1 (4%) | 2 (5%) |  |
| - Mixed | 3 (13%) | 2 (5%) |  |
| - Other | 0 | 3 (13%) |  |
| History of allergic reaction to egg   - Baked egg - Whole egg - Raw egg | 8 (35%)  20 (87%)  14 (61%) | 18 (49%)  28 (76%)  16 (43%) | 0.422  0.340  0.288 |
| Symptoms at last reaction to baked egg |  |  |  |
| - Erythematous pruritic rash | 4 (17%) | 8 (22%) | 0.752 |
| - Urticaria (>3 urticarial lesions) | 1 (4%) | 5 (14%) | 0.391 |
| - Angioedema (>1 site) | 4 (17%) | 7 (19%) | 1.0 |
| - Persistent rubbing of nose and eyes | 0 | 0 | N/A |
| - Persistent rhinorrhoea | 0 | 0 | N/A |
| - Persistent scratching | 2 (9%) | 1 (3%) | 0.552 |
| - Stridor | 0 | 0 | N/A |
| - Dysphonia | 0 | 1 (3%) | 1.0 |
| - Persistent cough | 1 (4%) | 0 | 0.383 |
| - Wheezing | 1 (4%) | 0 | 0.383 |
| - Severe abdominal pain | 2 (9%) | 4 (11%) | 1.0 |
| - Vomiting | 3 (13%) | 6 (16%) | 1.0 |
| - Diarrhoea | 0 | 2 (5%) | 0.519 |
| - Apathy / anxiety | 0 | 1 (3%) | 1.0 |
| - Hypotension | 0 | 0 | N/A |
| History of consumption of baked egg | 2/12 responders (17%) | 7/34 responders (17%) | 0.673 |
| High-risk dose given during DBPCFC | 9 (39%) | 15 (41%) | 1.0 |
| Atopic eczema | 21 (91%) | 27 (73%) | 0.107 |
| Other food allergies | 11 (73%) | 35 (78%) | 0.487 |
| Allergic rhinitis | 13 (57%) | 12 (32%) | 0.105 |
| Asthma | 9 (39%) | 15 (41%) | 1.0 |
| Time to reaction (min) | 12 (2.5; 23) | 17 (6; 24) | 0.184 |
| Cumulative dose tolerated (g) | 0.14 (0.03; 1.03) | 0.06 (0.01; 0.44) | 0.111 |
| Eliciting dose (g) | 0.3 (0.1; 1.0) | 0.3 (0.1; 0.6) | 0.338 |

Abbreviations: DBPCFC: Double-blind placebo-controlled food challenges.

**Table S5.** Demographic and clinical characteristics of baked egg allergic patients (n=60) who reacted to the median cumulative tolerated dose (0.13g of egg protein) or less and baked egg allergic patients who reacted to more than 0.13g of egg protein during OFC to baked egg in the BAT2 study. P values refer to Mann-Whitney U test.

| **Demographic and clinical characteristics** | **Threshold <=0.13g (n=33)** | **Threshold >0.13g (n=27)** | **P value** |
| --- | --- | --- | --- |
| Age (years) | 5 (2.5; 8.5) | 7.0 (2.5; 9) | 0.462 |
| Gender (% females) | 18 (55%) | 8 (30%) | 0.069 |
| Ethinicity |  |  |  |
| 1. White | 22 (67%) | 17 (63%) |  |
| 1. Black | 4 (12%) | 2 (7%) |  |
| 1. Asian | 2 (6%) | 2 (7%) | 0.902 |
| 1. Chinese | 2 (6%) | 1 (4%) |  |
| 1. Mixed | 2 (6%) | 3 (11%) |  |
| 1. Other | 1 (3%) | 2 (7%) |  |
| History of allergic reaction to egg   1. Baked egg 2. Whole egg 3. Raw egg | 17 (52%)  30 (91%)  20 (61%) | 9 (33%)  18 (67%)  10 (37%) | 0.196  0.026  0.119 |
| History of consumption of baked egg | 5/29 responders (17%) | 4/24 responders (17%) | 0.624 |
| High risk dose given | 13 (39%) | 11 (41%) | 1.0 |
| Atopic eczema | 23 (70%) | 25 (93%) | 0.049 |
| Other food allergies | 25 (76%) | 21 (78%) | 0.551 |
| Allergic rhinitis | 13 (39%) | 12 (44%) | 0.794 |
| Asthma | 11 (33%) | 13 (48%) | 0.295 |
| Symptom severity score | 3 (1; 3) | 3 (3; 3) | **0.008** |
| Time to reaction (min) | 15 (6; 22) | 16 (3; 30) | 0.851 |
| Cumulative dose tolerated (g) | 0.03 (0.01; 0.04) | 0.46 (0.23; 1.04) | **<0.001** |
| Eliciting dose (g) | 0.10 (0.03; 0.10) | 0.60 (0.30; 1.50) | **<0.001** |

**Table S**6**.** Basophil activation test to baked egg results for baked egg allergic patients (n=60) who developed severe or non-severe reactions during DBPCFC to baked egg, according to the Practall guidelines. P values refer to Mann-Whitney U test.

| **Basophil activation test** | **Severe reactors**  **(n=23)** | **Non-severe reactors (n=37)** | **P value** |
| --- | --- | --- | --- |
| BAT to egg at 0.1ng/ml (%CD63+ Basophils) | 1.3 (0; 17.3) | 1.3 (0.1; 2.6) | 0.816 |
| BAT to egg at 1ng/ml  (%CD63+ Basophils) | 3.5 (0.9; 20.7) | 2.3 (0.7; 9.0) | 0.328 |
| BAT to egg at 10ng/ml  (%CD63+ Basophils) | 14.0 (7.2; 44.6) | 7.1 (2.6; 25.0) | 0.076 |
| BAT to egg at 100ng/ml  (%CD63+ Basophils) | 28.4 (16.1; 53.2) | 15.0 (7.0; 42.3) | 0.060 |
| BAT to egg at 1000ng/ml (%CD63+ Basophils) | 28.6 (8.7; 52.7) | 17.4 (7.4; 30.3) | 0.068 |
| BAT to egg at 10000ng/ml (%CD63+ Basophils) | 21.9 (11.2; 53.5) | 16.8 (5.1; 31.7) | 0.093 |
| BAT to baked egg at 0.1ng/ml (%CD63+ Basophils) | 9.2 (1.1; 34.9) | 1.0 (0; 4.2) | **0.001** |
| BAT to baked egg white at 1ng/ml  (%CD63+ Basophils) | 16.6 (1.2; 31.5) | 2.2 (0; 9.8) | **0.003** |
| BAT to baked egg white at 10ng/ml  (%CD63+ Basophils) | 24.2 (2.2; 48.0) | 5.3 (0.2; 17.8) | **0.005** |
| BAT to baked egg white at 100ng/ml  (%CD63+ Basophils) | 27.7 (8.7; 47.6) | 7.4 (0; 21.4) | **0.003** |
| BAT to baked egg white at 1000ng/ml (%CD63+ Basophils) | 27.7 (8.7; 47.6) | 5.8 (0.1; 21.1) | **0.017** |
| BAT to baked egg white at 10000ng/ml (%CD63+ Basophils) | 29.4 (5.7; 42.6) | 6.1 (0.4; 24.7) | **0.043** |
| BAT to egg at 0.1ng/ml  (SI CD203c) | 1.3 (1.0; 2.3) | 1.1 (1.0; 1.4) | 0.231 |
| BAT to egg at 1ng/ml  (SI CD203c) | 1.7 (1.2; 3.1) | 1.4 (1.1; 2.0) | 0.257 |
| BAT to egg at 10ng/ml  (SI CD203c) | 2.9 (1.4; 4.3) | 1.8 (1.3; 4.1) | 0.376 |
| BAT to egg at 100ng/ml  (SI CD203c) | 4.0 (2.2; 5.9) | 2.6 (1.9; 4.6) | 0.148 |
| BAT to egg at 1000ng/ml  (SI CD203c) | 4.1 (2.3; 6.1) | 2.8 (1.9; 4.1) | 0.176 |
| BAT to egg at 10,000ng/ml  (SI CD203c) | 3.6 (2.0; 4.9) | 2.7 (1.8; 4.4) | 0.367 |
| BAT to baked egg white at 0.1ng/ml  (SI CD203c) | 1.8 (1.3; 3.6) | 1.1 (1.0; 1.6) | **0.019** |
| BAT to baked egg white at 1ng/ml  (SI CD203c) | 2.5 (1.3; 3.9) | 1.2 (1.1; 2.1) | **0.010** |
| BAT to baked egg white at 10ng/ml  (SI CD203c) | 3.4 (1.9; 4.8) | 1.7 (1.1; 2.9) | **0.039** |
| BAT to baked egg white at 100ng/ml  (SI CD203c) | 3.6 (2.0; 5.2) | 2.1 (1.0; 3.5) | **0.032** |
| BAT to baked egg white at 1000ng/ml  (SI CD203c) | 3.8 (1.7; 5.6) | 2.1 (1.1; 3.6) | **0.025** |
| BAT to baked egg white at 10,000ng/ml  (SI CD203c) | 3.2 (1.3; 5.9) | 2.1 (1.1; 3.7) | 0.093 |

Abbreviations: Basophil activation test (BAT); DBPCFC: Double-blind placebo-controlled food challenges.

**Table S7.** Basophil activation test to baked egg results for baked egg allergic patients (n=60) with lower versus higher threshold of reactivity to baked egg during DBPCFC to baked egg. P values refer to Mann-Whitney U test.

| **Basophil activation test** | **Threshold <=0.13g (n=33)** | **Threshold >0.13g (n=27)** | **P value** |
| --- | --- | --- | --- |
| BAT to egg at 0.1ng/ml (%CD63+ Basophils) | 1.7 (0.3; 15.9) | 0.7 (0; 2.8) | 0.063 |
| BAT to egg at 1ng/ml  (%CD63+ Basophils) | 4.3 (1.2; 25.9) | 2.2 (0; 11.6) | 0.052 |
| BAT to egg at 10ng/ml  (%CD63+ Basophils) | 14.9 (4.2; 44.6) | 7.2 (2.3; 19.6) | 0.112 |
| BAT to egg at 100ng/ml  (%CD63+ Basophils) | 17.5 (9.5; 51.7) | 20.8 (6.3; 35.2) | 0.488 |
| BAT to egg at 1000ng/ml (%CD63+ Basophils) | 18.2 (8.1; 42.4) | 21.5 (7.2; 41.0) | 0.723 |
| BAT to egg at 10000ng/ml (%CD63+ Basophils) | 18.6 (5.6; 44.6) | 19.4 (6.3; 38.7) | 0.960 |
| BAT to baked egg at 0.1ng/ml (%CD63+ Basophils) | 4.3 (0.7; 17.9) | 1.2 (0; 8.7) | 0.146 |
| BAT to baked egg white at 1ng/ml  (%CD63+ Basophils) | 9.6 (1.0; 21.9) | 2.7 (0; 16.7) | 0.265 |
| BAT to baked egg white at 10ng/ml  (%CD63+ Basophils) | 12.5 (1.2; 42.1) | 13.5 (0.9; 27.1) | 0.685 |
| BAT to baked egg white at 100ng/ml  (%CD63+ Basophils) | 12.0 (1.5; 35.0) | 20.1 (0.5; 35.6) | 0.852 |
| BAT to baked egg white at 1000ng/ml (%CD63+ Basophils) | 8.6 (0.3; 34.9) | 21.1 (0.9; 36.6) | 0.673 |
| BAT to baked egg white at 10000ng/ml (%CD63+ Basophils) | 12.3 (1.2; 35.9) | 17.8 (1.6; 36.2) | 0.946 |
| BAT to egg at 0.1ng/ml  (SI CD203c) | 1.3 (1.0; 3.0) | 1.0 (1.0; 1.4) | 0.063 |
| BAT to egg at 1ng/ml  (SI CD203c) | 1.8 (1.4; 4.2) | 1.2 (1.1.;1.8) | **0.008** |
| BAT to egg at 10ng/ml  (SI CD203c) | 3.4 (1.9; 5.5) | 1.7 (1.4; 2.8) | **0.003** |
| BAT to egg at 100ng/ml  (SI CD203c) | 4.0 (2.6; 6.5) | 2.5 (2.1; 4.6) | 0.068 |
| BAT to egg at 1000ng/ml  (SI CD203c) | 3.4 (2.5; 5.7) | 3.0 (2.3; 4.5) | 0.287 |
| BAT to egg at 10,000ng/ml  (SI CD203c) | 3.1 (2.0; 5.7) | 2.7 (1.9; 4.4) | 0.417 |
| BAT to baked egg white at 0.1ng/ml  (SI CD203c) | 1.6 (1.2;3.4) | 1.0 (1.0; 1.3) | **0.019** |
| BAT to baked egg white at 1ng/ml  (SI CD203c) | 2.0 (1.1; 3.9) | 1.2 (1.1; 1.6) | 0.065 |
| BAT to baked egg white at 10ng/ml  (SI CD203c) | 2.9 (1.2; 4.7) | 1.7 (1.3; 2.7) | 0.108 |
| BAT to baked egg white at 100ng/ml  (SI CD203c) | 2.8 (1.1; 5.0) | 2.5 (1.9; 4.5) | 0.532 |
| BAT to baked egg white at 1000ng/ml  (SI CD203c) | 2.5 (1.3; 4.5) | 2.9 (2.2; 4.5) | 0.866 |
| BAT to baked egg white at 10,000ng/ml  (SI CD203c) | 2.5 (1.3; 5.1) | 2.5 (1.8; 4.3) | 0.612 |

Abbreviations: Basophil activation test (BAT); DBPCFC: Double-blind placebo-controlled food challenges.

**Figure S1.** Criteria for stopping the oral food challenge followed the Practall guidelines.


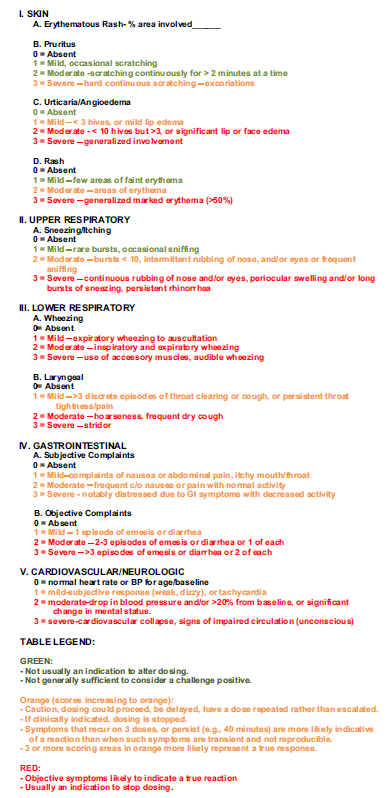


**Figure S2.** Basophil activation to baked egg and egg extract as well as controls in (A) severity and (B) threshold groups, using CD63 and CD203c as activation markers, respectively. Red, severe/low threshold and blue, non-severe/high threshold.

**A.**

**B.**

**Figure S3.** Proposed practical application of BAT to inform clinical decision making on diagnosis and prognosis in terms of severity and threshold of allergic reactions to baked egg and loosely cooked egg, which should be integrated into the clinical and wider context of individual patients. 3


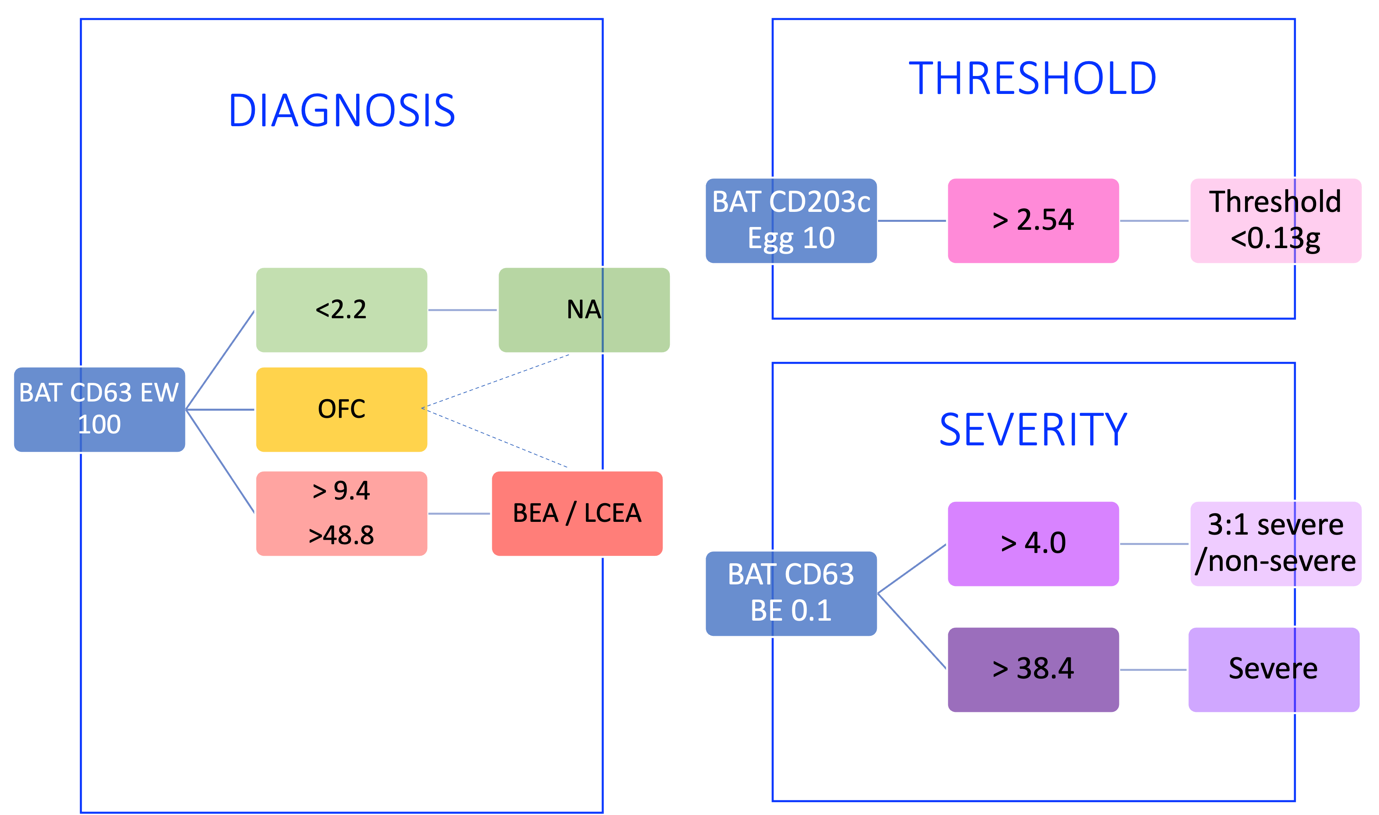

Supplement: Supplementary file 1 — Appendix S1 [file ALL-79-419-s001.docx]
